# Self-compassion is associated with the superior longitudinal fasciculus in the mirroring network in healthy individuals

**DOI:** 10.1192/j.eurpsy.2023.1160

**Published:** 2023-07-19

**Authors:** M.-K. Kim, Y.-G. Hwang, C. Pae, C. R. Song, M. Bang, C. I. Park, S.-H. Lee

**Affiliations:** 1 Department of Psychiatry, CHA Ilsan Medical Center, CHA University, Goyang-si, Gyeonggi-do; 2 Department of Psychiatry, CHA Bundang Medical Center, CHA University, Seongnam, Korea, Republic Of

## Abstract

**Introduction:**

Self-compassion (SC) describes an emotionally positive attitude extended toward ourselves when we suffer, consisting of three main components; self-kindness, common humanity, and mindfulness (Germer & Neff, 2013). SC entails being warm and understanding towards ourselves when encountering pain or personal shortcomings, rather than ignoring them or flagellating ourselves with self-criticism. SC also involves recognizing that suffering and failure are part of the shared human experience rather than isolating. In addition, SC requires taking a mindful approach to one’s feelings and thoughts, without judgment of them.

**Objectives:**

Self-compassion (SC) involves taking an emotionally positive attitude towards oneself when suffering. Although SC has positive effects on mental well-being as well as a protective role in preventing depression and anxiety in healthy individuals, few studies on white matter (WM) microstructures in neuroimaging studies of SC has been studied.

**Methods:**

Magnetic resonance imaging data were acquired from 71 healthy participants with measured levels of SC and its six subscales. Mirroring network as WM regions of interest were analyzed using tract-based spatial statistics (TBSS). After the WM regions associated with SC were extracted, exploratory correlation analysis with the self-forgiveness scale, the coping scale, and the world health organization quality of life scale abbreviated version was performed.

**Results:**

We found that self-compassion scale (SCS) total scores were negatively correlated with the fractional anisotropy (FA) values of the superior longitudinal fasciculus (SLF) in healthy individuals. The self-kindness and mindfulness subscale scores of SCS were also negatively correlated with FA values of the same regions. The FA values of SLF related to SC were found to be negatively correlated with the total scores of self-forgiveness scale, and self-control coping strategy and confrontation coping strategy.

**Conclusions:**

Our findings suggest that levels of SC and its self-kindness and mindfulness components may be negatively associated with DMN-related WM microstructures in healthy individuals. These less WM microstructures may be associated with positive personal attitudes, such as self-forgiveness, self-control and active confrontational strategies.

**Disclosure of Interest:**

None Declared

